# The Mechanism of Effective Electroacupuncture on T Cell Response in Rats with Experimental Autoimmune Encephalomyelitis

**DOI:** 10.1371/journal.pone.0051573

**Published:** 2013-01-28

**Authors:** Yumei Liu, Hongwei Wang, Xinyue Wang, Lili Mu, Qingfei Kong, Dandan Wang, Jinghua Wang, Yao Zhang, Jinfeng Yang, Mingyan Zhou, Guangyou Wang, Bo Sun, Hulun Li

**Affiliations:** 1 Department of Neurobiology, Harbin Medical University, Heilongjiang Provincial Key Laboratory of Neurobiology, Harbin, China; 2 Department of Orthopaedics, the Second Affiliated Hospital of Harbin Medical University, Harbin, China; 3 The Key Laboratory of Myocardial Ischemia, Chinese Ministry of Education, Harbin Medical University, Harbin, China; Washington University, United States of America

## Abstract

Previously, we demonstrated that electroacupuncture (EA) decreased lymphocyte infiltration into the spinal cords of rats presenting with experimental autoimmune encephalomyelitis (EAE), a disease model used in the study of multiple sclerosis (MS). The aim of this study was to characterize the effects of EA on the EAE. Female Lewis rats were divided into either CFA, EAE, EA, or injection with naloxone after electroacupuncture (NAL) groups. Electroacupuncture was administered every day for 21 days. To evaluate proliferation and apoptosis, lymphocytes from rats presenting with EAE were collected and cultured with β-endorphin. Immunohistochemisty, flow cytometry and radio-immunity methods were applied to detect the expression of β-endorphin. Results presented in this report demonstrate that the beneficial anti-inflammatory effects of EA on EAE were related to β-endorphin production that balances the Thl/Th2 and Th17/Treg responses. These results suggest that β-endorphin could be an important component in the development of EA-based therapies used for the treatment of EAE.

## Introduction

Multiple sclerosis (MS) is a disease that affects the central nervous system (CNS). The animal model used to study MS is experimental autoimmune encephalomyelitis (EAE) that mimics many of the clinical and pathological features of MS.

Gironi *et al*. found a reduction in β-endorphin (EB) levels in peripheral blood mononuclear cells (PBMCs) from patients with clinically inactive MS [1]. In 1973, endorphins (including enkephalins) were identified and these peptides were not only shown to act in an antinociceptive manner but also in ameliorating synovitis associated with rheumatoid arthritis and down-regulation of the inflammatory process [2], [3]. β-endorphine (BE) is a 31 amino acid opioid peptide synthesized primarily by the arcuate nucleus of the hypothalamus. A second β-endorphin system is found in the anterior pituitary where β-endorphin is co-released with ACTH (adrenocorticotropic hormone) into the bloodstream and exerts its effects on different target organs [4]. Studies in humans and animals indicated that BE exerted a physiological inhibitory effect on immune function [5] and BE has been shown shift the immune response from Th1 to Th2 response [6], [7].

Early studies showed that administration of the opiate receptor antagonist naloxone to either humans or animals increased T cell proliferative responses, up-regulated IL-2 and IFN-γ production while decreasing IL-4 production [8], [9]. Also, various studies have shown that immune cells can synthesize and secrete the opioid peptides [10]–[12] and leukocytes expressing opioid receptors have been shown to synthesize and release β-endorphin under certain conditions [13], [14]. Furthermore, some research has shown opioid-mediated modulation of proliferative responses, cytokine production profiles, and chemotactic potential of these cell types [15]–[19].

Acupuncture has long been practiced in China and in Western countries represents one of the most popular forms of alternative medicine. Recently, our laboratory demonstrated that electroacupuncture (EA) stimulation relieved EAE severity compared to no treatment or non-Zusanli-acupoint groups and modulated the immune response by increasing production of ACTH by the hypothalamus in EAE rats [20]. However, the exact mechanism(s) associated with EA-mediated immunomodulation have not been clearly defined. Acupuncture research has proved that stimulation of acupoints may release endorphins responsible amelioration of pain and cardiovascular diseases [21], [22]. Previous work by our group demonstrated that stimulation of the Zusanli acupoints (ST36) released endorphins associated with reduction in EAE severity by modulating immune cells.

The present study was undertaken to determine if rats with EAE presented with alterations in β-endorphin production and immune system function post EA treatment. To investigate this, we used *in vivo* and *in vitro* approaches. *In vivo*, we examined the effect of repeated EA treatments on the β-endorphin concentrations in the hypothalamus and plasma and the effect on immune cell populations after lymphocytes were co-cultured with β-endorphin *in vitro*.

## Materials and Methods

### Rats and Ethics Statement

Eight-week-old female Lewis rats were obtained from the Peking Vital River Laboratory Animal Ltd. (Peking, China) as previously described [20]. Animals had free access to food and water and maintained in sterile microisolator cages under specific-pathogen-free conditions at 21±2°C and 50±5% humidity and all rats were under ether anesthesia before they were sacrificed. All animal handling and experimental procedures were performed according to the guidelines established by the Care and Use of Laboratory Animals published by the China National Institute of Health (HMUIRB20120005) and approved by the Institutional Review Board of Harbin Medical University.

### Reagents

The myelin basic protein (MBP_68–86_) (YGSLPQKSQRSQDENPV) peptide was synthesized at AC Scientific, Inc. (Xian, China). A synthetic peptide corresponding to region α97–116 of the rat AChR (DGDFAIVKFTKVLLDYTGHI) was synthesized by AC Scientific, Inc. *Mycobacterium tuberculosis* (strain H37RA) was obtained from Difco (Detroit, MI) and emulsified with incomplete Freund’s adjuvant (Sigma, St. Louis, MO) to generate complete Freund’s adjuvant (CFA). The following antibodies were purchased from commercially available sources: fluorescein isothiocyanate (FITC)-conjugated anti-rat CD4 and phycoerythrin (PE)-conjugated anti-rat Foxp3 (eBioscience, San Diego, CA); PE-conjugated anti-rat IFN-γ; PE-conjugated anti-rat IL-4 (BD Biosciences, San Jose, CA); Cy3-conjugated goat anti-rabbit immunoglobulin G (IgG) (Caltag Laboratories, Burlingame, CA); PE-conjugated donkey anti-goat immunoglobulin G (IgG) (Abcam, CA). Rabbit polyclonal anti-IL-17 and goat polyclonal anti-β-endorphin were purchased from Santa Cruz Biotechnologies (Santa Cruz, CA).

### Treatment Groups

Animals were divided into 4 treatment groups: (1) CFA emulsified in phosphate buffered saline (PBS) (CFA contained *M. tuberculosis* strain R37RA at a concentration of 20 mg/ml), (2) the EAE group consisted of rats immunized subcutaneously in the tail with 0.2 ml of 0.025 mg MBP_68–86_ emulsified in CFA, (3) the Zusanli acupoint (EA) immunization group that was immunized as group 2 but treated with EA, and (4) the NAL group that consisted of animals injected with naloxone (0.4 mg/kg) intravenously after electroacupuncture in 30 min. Prior to delivery, naloxone was diluted in sterile saline so that a 100 µl injection contained 250 µg of the drug. The Zusanli acupoint (ST36) is located 5 mm ventral and lateral to the anterior tubercle of the tibia. EA stimulation was applied for 30 min, started on the day of immunization, and repeated each day for a period of 21 days. Rats were scored for EAE as follows: 0, no disease; 1, piloerection; 2, loss in tail tonicity; 3, hind leg paralysis; 4, paraplegia, and 5, moribund or dead. Mean clinical scores at separate days and mean maximal scores were calculated by adding scores of individual rats and dividing by number of rats in each group.

### Apoptosis Assessment

Apoptotic lymphocytes were identified by characteristic morphological changes and expressed as a percentage of total lymphocytes counted. EAE, EA, and NAL group rats were sacrificed 7, 14 and 21 days post primary immunization and lymphocytes harvested. Besides, the cells from 14 days were cultured with 10^−8^ M β-endorphin or 10^−8^ M β-endorphin and 10^−4^ M naloxone. And then apoptosis was analyzed by flow cytometry following Annexin V (AV) and propidium iodide (PI) labeling following the manufacturer’s staining protocol (NeoBioscience, Shenzhen, China). Analysis was performed using Cell Quest Pro Software (BD Biosciences). Samples were gated on the granulocyte population using forward and side scatter plots with a minimum of 10,000 gated events sampled. Cells were defined as apoptotic (AV^+^/PI^−^), secondary necrotic (AV^+^/PI^+^), necrotic (AV^+^/PI^−^), or neither (AV^−/^PI^−^). Each subpopulation was expressed as a percentage of the total population of granulocytes.

### Immunohistochemistry

Frozen spleen sections from EAE rats on 14 day immunization were stained with goat anti-rat β-endorphin followed by a horseradish peroxidase-labeled anti-goat secondary antibody and 3, 30-Diaminobenzidine (DAB) substrate to detect β-endorphin expression. The number of positive-staining cells was measured from digital images using IMAGE PRO PLUS software (Media Cybernetics, Silver Springs, MD).

### T-cell Proliferation Assay

Triplicate aliquots (200 µl) of lymphocyte suspensions containing 4×10^5^ cells were placed in 96-well, round-bottom microtitre plates, and stimulated with MBP_68–86_ (20 µg/ml), MBP_68–86_ peptides (20 µg/ml)+β-endorphin (10^−8^ M), MBP_68–86_ (10 µg/ml)+β-endorphin (10^−7^ M), MBP_68–86_ (10 µg/ml)+β-endorphin (10^−6^ M), MBP_68-86_ peptides (20 µg/ml)+β-endorphin (10^−8^ M)+naloxone (10^−4^ M) or PBS. Stimulation with concanavalin A (5 µg/ml) was used as a positive control. After a 54 h incubation, cells were pulsed for another 18 h with 10 µl PBS containing 1 µCi [^3^H] methylthymidine (specific activity 60 Ci/mmol; China Institute of Atomic Energy, Beijing, China), and results expressed as mean counts per minute of triplicate cultures.

### Immunofluorescent Staining for Flow Cytometry

EAE, EA, and NAL group rats were sacrificed 14 days post primary immunization and lymphocytes harvested. To evaluate CD4^+^ T cell profile distribution and the β-endorphin expression levels we performed standard flow cytometric assays. Brefeldin A (1∶1000 dilution) (eBioscience), a protein transport inhibitor preventing cytokine secretion, was added to the cell culture media and incubated for 5 h. After washing twice with staining buffer, cells were stained extracellularly with FITC-conjugated anti-CD4. After fixation and permeabilization, cells were stained intracellularly with PE-conjugated anti-rat-IFN-γ (BD Biosciences), anti-rat-IL-4 (BD Biosciences), anti-rat-Foxp3 (BD Biosciences), rabbit anti-IL-17 (Santa Cruz Biotechnology) followed by Cy3-conjugated goat anti–rabbit IgG (Caltag Laboratories) or goat anti-β-endorphin (Santa Cruz Biotechnology) followed by PE-conjugated anti-goat IgG (Abcam). Samples were tested using a FACSCalibur flow cytometer and data analyzed with Cell Quest Pro Software (BD Biosciences).

### Determination of Plasma and Hypothalamus β-endorphin Concentrations

Rats from respective treatment groups were sacrificed 7, 14, and 21 d post-MBP_68–86_ immunization under ether anesthesia and blood from 5 rats/group was obtained by cardiac puncture and collected in heparinized tubes containing sodium EDTA. Plasma was separated by centrifugation at 4°C and stored at −80°C until use. β-endorphin samples from the hypothalamus were extracted as described by Javadi *et al*. (Javadi, 2003) and stored at −80°C until analyses were performed. Plasma and hypothalamus β-endorphin concentrations were determined using the β-endorphin^ 125^I RIA kit (Tianjin Hope Year Medical Products Co., Ltd., China). The β-endorphin assay sensitivities were 3.5 pg/ml.

### Statistical Analysis

Data are expressed as the mean ± standard error (SE) of 4–8 observations. Statistical analyses were performed using the SPSS (Statistical Package for Social Sciences, Chicago, IL) software. One-way analysis of variance (ANOVA) was used to determine statistical differences between groups. Clinical scores were analyzed using the non-parametric Mann–Whitney U-test. *P*<0.05 was considered statistically significant.

## Results

### Effect of Naloxone Post Electroacupuncture Treatment of EAE

**Figure 1:**
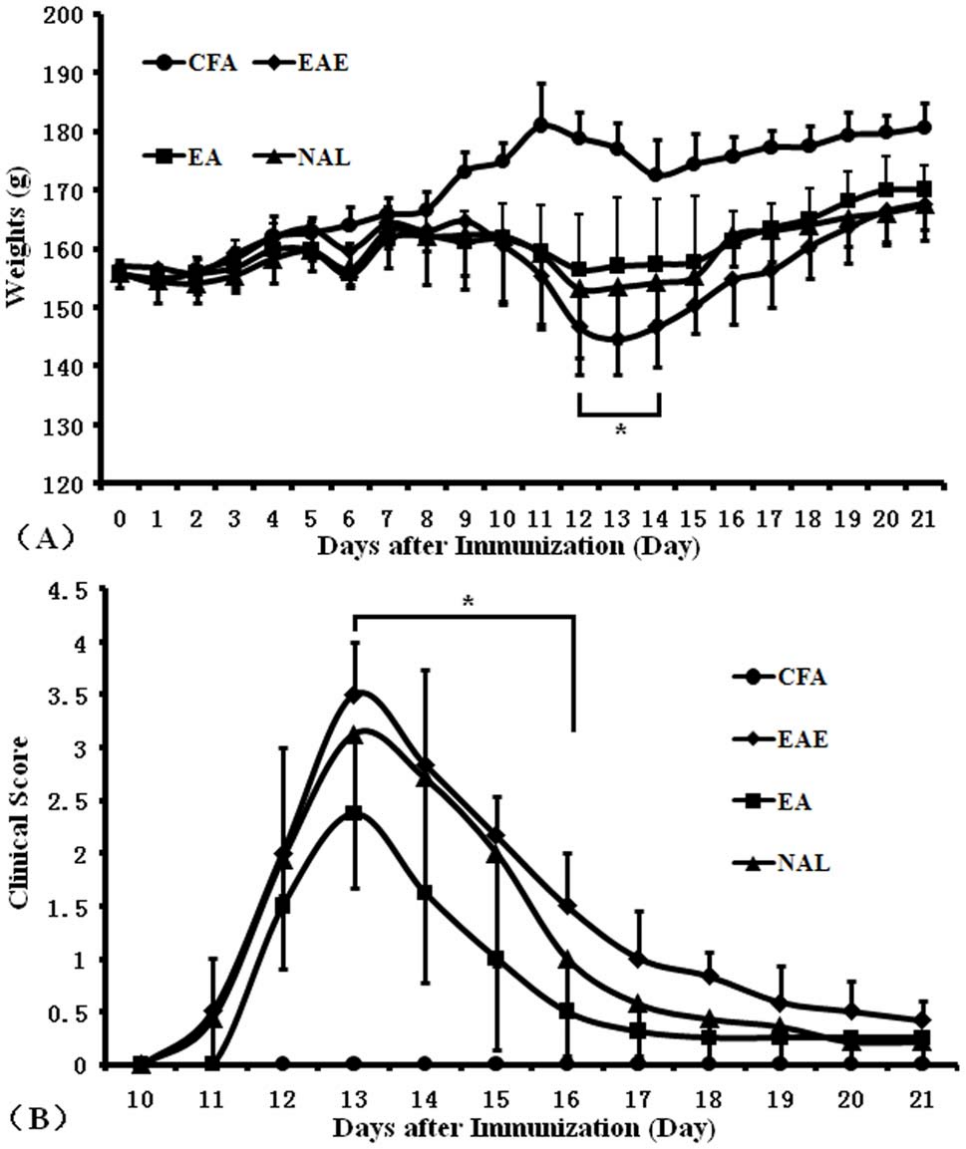
Body weight and clinical scores. After the first immunization, mice were weighed and disease severity scored daily until day 21 post immunization. Body weights (A) and clinical scores (B) were measured in control rats (•), EAE rats (♦), EA rats (▪), and NAL rats (▴) (n = 8 for EAE and NAL groups, n = 9 for EA groups, n = 5 for the CFA group, *P<0.05). The results shown are representative of 3 separate experiments.

We previously determined that electroacupuncture treatment of rats decreased lymphocyte infiltration into spinal cords and reduced EAE severity in rats (20). Similar results also showed that rats in the EAE group lost significant weight compared to animals in the EA group by days 12–14 days post-immunization (Fig. 1B, *P*<0.05) and rats treated with EA 13, 14 and, 15 days post EAE induction presented with reduced EAE clinical scores compared to untreated rats (Fig. 1B, *P*<0.05). We focused on analysis of the NAL group and demonstrated that despite electroacupuncture administration, EAE presentation in this group was similar to presentation in rats in the EAE group. These facts suggested acupuncture perhaps could enhance endogenous opioid peptides in EAE rats and the effects of beta-endorphins on EAE.

### β-endorphin-mediated Inhibition of MBP-specific T Cell Proliferation

**Figure 2:**
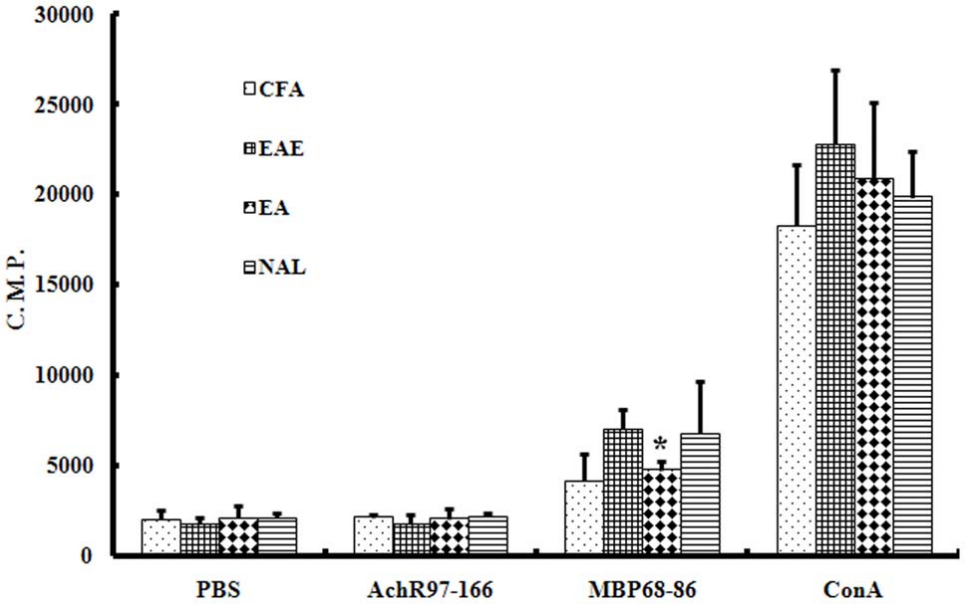
The effect of EA treatment on lymphocyte proliferation. Lymphocytes were isolated from CFA, EAE, EA, and, NAL rats 14 days post immunization. Cells were incubated with or without MBP_68–86_ (10 mg/ml) or AchR_97–166_ (10 µg/ml) or ConA (5 mg/ml) for 48 h. Cell proliferation was assessed by [^3^H]thymidine incorporation. Proliferation of lymphocytes from EA-treated rats was reduced. The results are shown as mean counts per minute (C.P.M.) ± SE. *P<0.05 vs. EAE group.

As our previous findings suggested, the level of proliferation of T cells harvested from EA group rats in response to MBP_68–86_ was reduced compared to lymphocytes harvested from EAE rats. T cells were harvested from rats in the CFA, EAE, EA, and NAL group rats and proliferation assessed by measuring [^3^H] thymidine incorporation (Fig. 2). These data supported previous observations demonstrating that the proliferation MBP_68–86_-specific T cells harvested from EA-treated rats was significantly reduced (*P*<0.05). There were no differences in proliferation between T cells harvested from NAL and EAE group rats.

**Figure 3:**
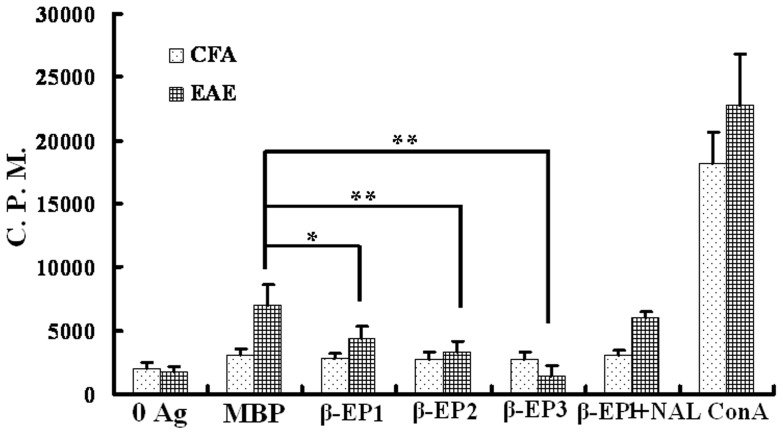
β-endorphin inhibits antigen-dependent proliferation of lymphocytes from EAE rats. Proliferation of rat CD4^+^ MBP specific T cells or CD4^+^ non-specific T cells stimulated with or without antigen in the absence or presence of different concentrations of β-endorphin and/or naloxone was assessed. β-EP1∶10^−8^ M β-endorphin, β-EP2∶10^−7^ M β-endorphin, β-EP3∶10^−6^ M β-endorphin, β-EP1+NAL: 10^−8^ M β-endorphin+10^−4^ M naloxone, *P<0.05, **P<0.01.

We next evaluated T cell proliferation by measuring [^3^H] incorporation 14 days post EAE induction in response to stimulation with either MBP_68–86_ peptides, MBP_68–86_ peptides+β-endorphin, or MBP_68–86_ peptides+β-endorphin+naloxone. This analysis demonstrated that T cells from EAE rats proliferated significantly more compared to proliferation observed in T cells treated with β-endorphin (Fig. 3) and that this effect was reversed by the naloxone.

### Quantification of Lymphocyte Apoptosis

**Figure 4:**
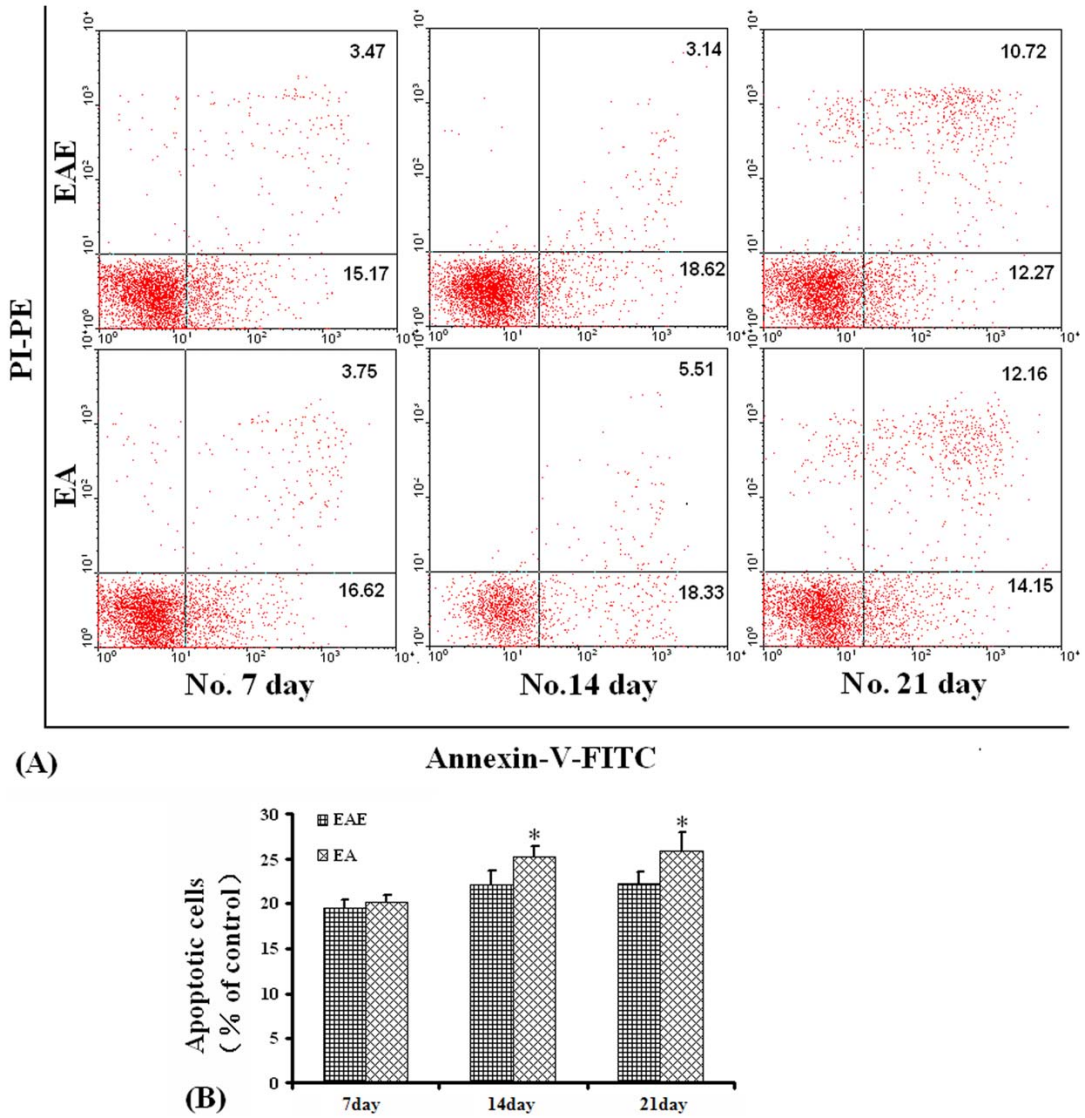
Apoptosis measurements. Apoptosis was determined by flow cytometric analysis using double staining of cells with Annexin V/PI. (A). Representative flow cytometric analysis of cells harvested from rats in the EAE and EA groups. (B). Percent number of cells undergoing apoptosis in rats from the EAE and EA groups over time. *P<0.05.

**Figure 5:**
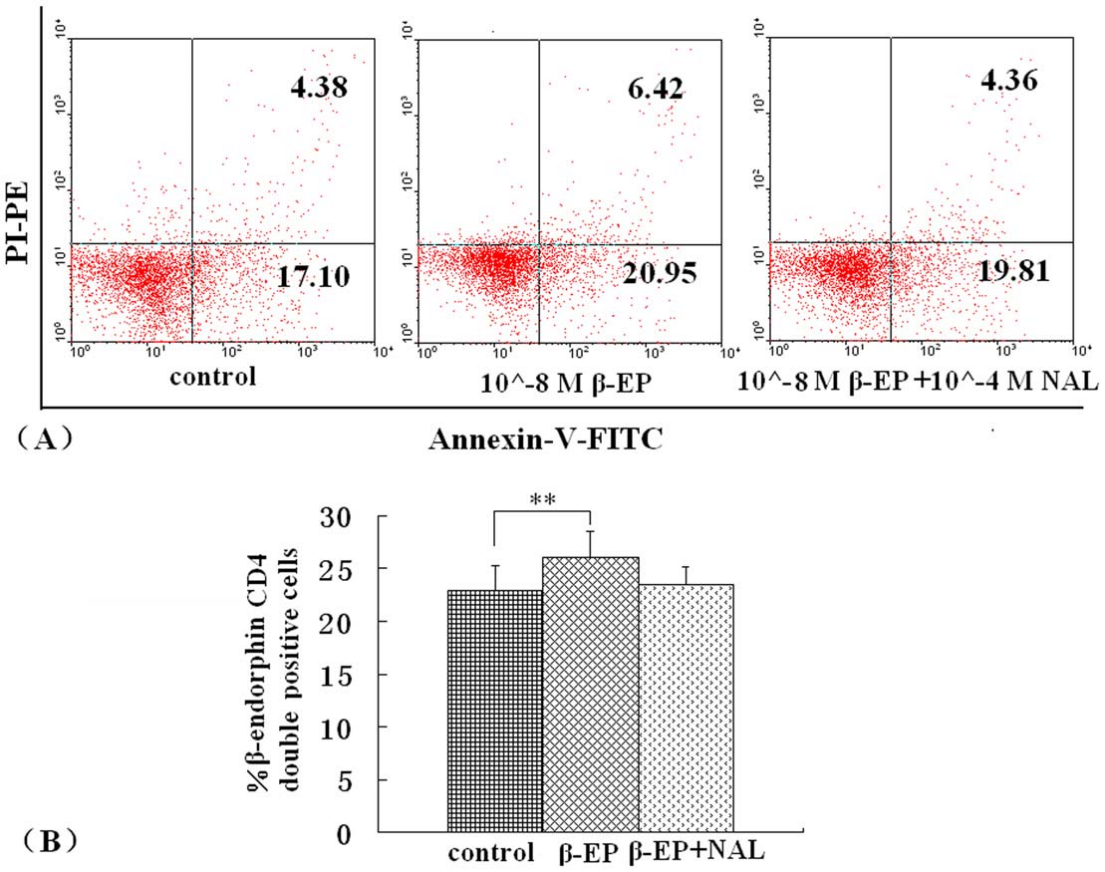
Effect of β-endorphin on lymphocytes apoptosis. Lymphocytes were harvested from EAE and cultured with 10^−8^ M β-endorphin. To detect apoptotic lymphocytes flow cytometric analysis was applied. (A). Representative flow cytometric analysis of apoptotic cells. (B). Percent number of cells undergoing apoptosis in the EAE lymphocytes, cultured with β-endorphin or β-endorphin and nalxone. **P<0.01 control vs. 10^−8^ M β-endorphin.

Flow cytometric assessment of apoptosis is summarized in Fig. 4 and Fig. 5. The percentage of apoptotic cells was significantly different between the EA and EAE groups 14 and 21 days post immunization (*P*<0.05), AV^+^/PI^+^, and AV^+^/PI^−^ cells compared to the percentage of lymphocytes presenting with apoptotic morphology (Fig. 4).

To examine the lymphocytes apoptosis treated with β-endorphin, lymphocytes from rats in the respective treatment groups were harvested on days 14 post immunization and cultured for 6–8 h in 10^∧^-8 M β-endorphin. Also there was a higher percentage of apoptotic lymphocytes detected by the flow cytometric assessment compared with those cells untreated (*P*<0.01) (Fig. 5).

### Effect of the ST36 Acupoint on β-endorphin Production

**Figure 6:**
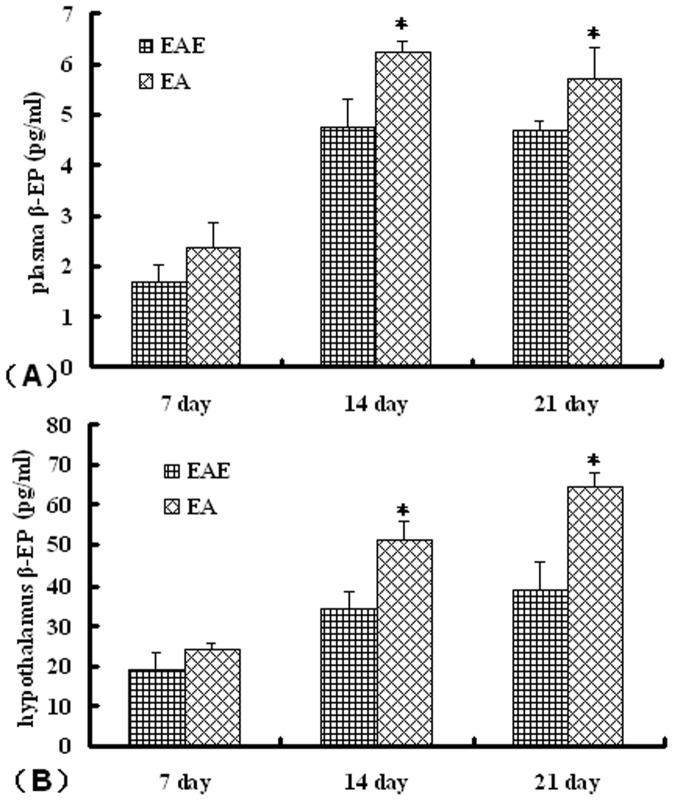
Effect of EA on β-endorphin levels in EAE rats. Rats from the respective groups were sacrificed at 7, 14, or 21 d post-immunization with MBP_68–86_ and the β-endorphin levels present in (A) plasma and (B) hypothalamus determined. Values are expressed as the mean ± SE of x observations (n = 5, *P<0.05, **P<0.01 EA vs. EAE group).

We examined the effects of EA on β-endorphin levels in rats with EAE. This analysis demonstrated that EA-treated rats had significantly elevated β-endorphin concentrations in both the hypothalamus and in plasma compared to untreated rats in the EAE group (Fig. 6).

**Figure 7:**
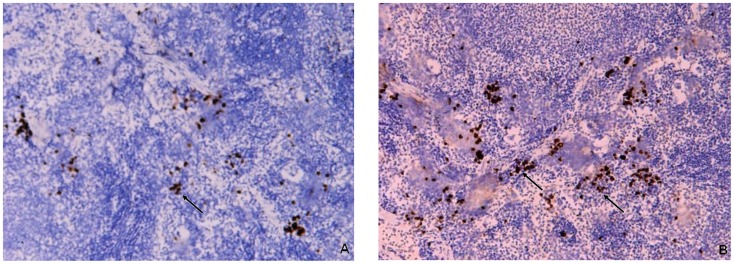
Expression of β-endorphin in spleen tissue sections. Immunohistochemical staining of β-endorphin positive cells in rat splenic tissues from EAE and EA rats 14 days post immunization. (A) β-endorphin positive cells in EAE rats. (B) β-endorphin positive cells in EA rats. Magnification: ×100.

Rat spleens were harvested and analyzed as indicated in the Materials and Methods for the detection of β-endorphin by immunohistochemistry. The expression of β-endorphin in the spleens of EA rats was significantly increased compared to levels observed in EAE group (Fig. 7).

**Figure 8:**
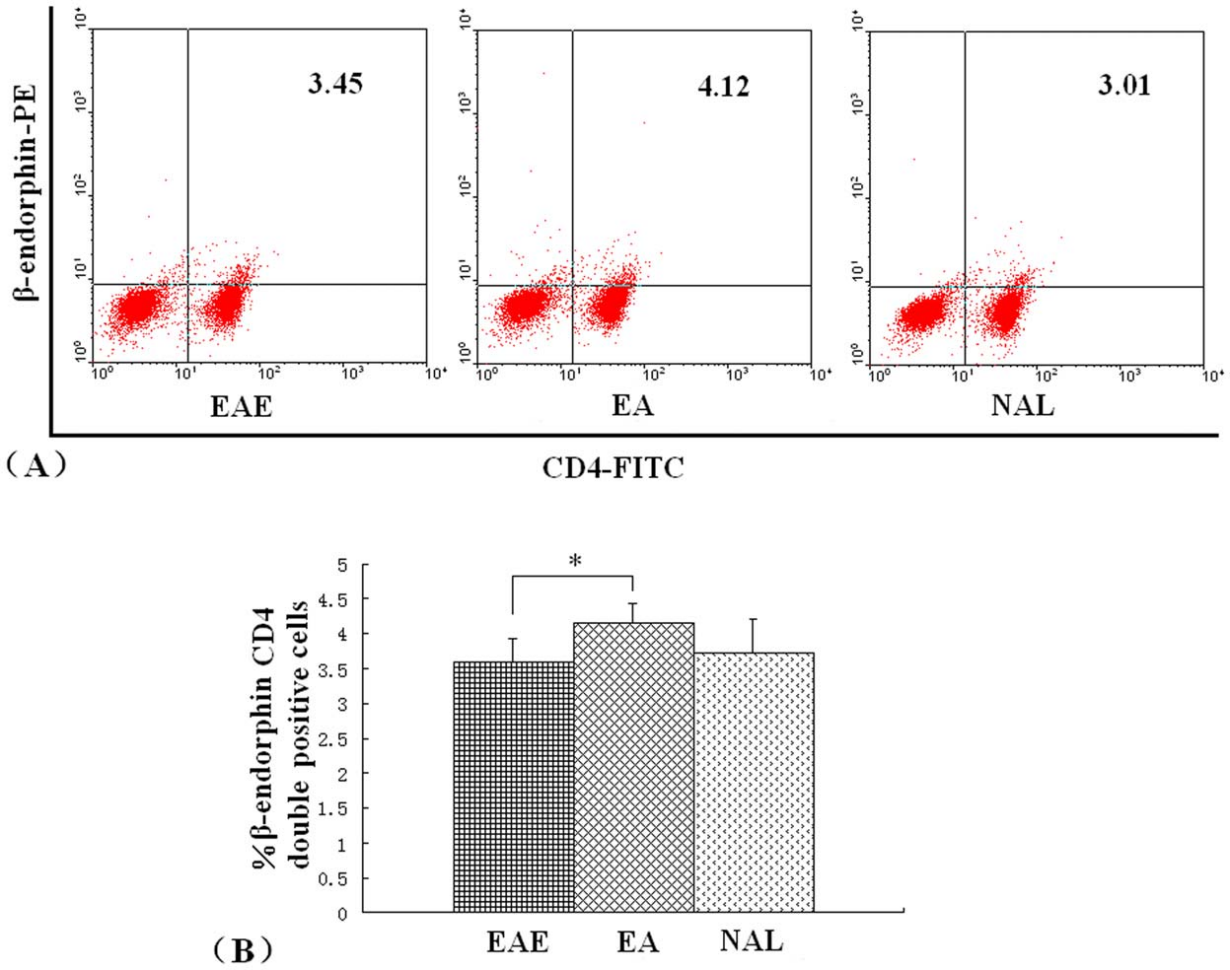
Effect of EA on CD4^+^ T cell β-endorphin expression in EAE rats. Lymphocytes from either EAE, EA, or NAL rat groups were isolated on day 14 post immunization. CD4^+^ cell expression of β-endorphin was assessed by flow cytometry. (A). Representative flow cytometric analysis of cells harvested from rats in the EAE, EA and NAL groups. (B). Percent number of CD4^+^ T cells β-endorphin expression in rats from the EAE, EA and NAL groups. *P<0.05.

There was a significant increase in the production of β-endorphin by CD4^+^ T cells in the EA group. Analysis of β-endorphin by CD4^+^ lymphocytes harvested 14 days post immunization from rats in the EAE, EA, or NAL groups was assessed by flow cytometry. The percentage of CD4^+^ T cells identified in EA rats was higher than the levels observed in cells harvested from EAE and NAL rats (Fig. 8).

### Effect of β-endorphin on EAE CD4^+^ T Cell Subsets

**Figure 9:**
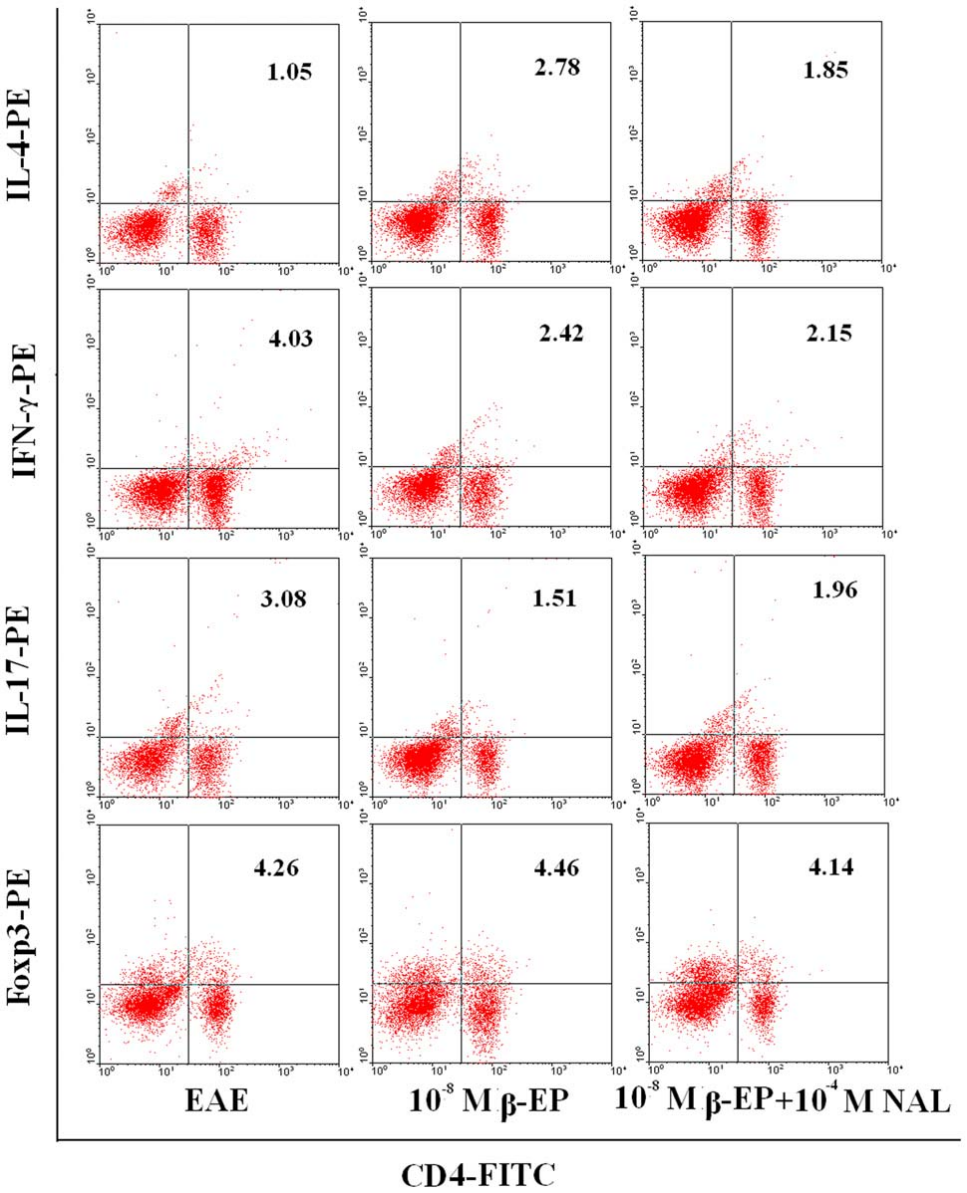
Subtype changes in MBP_68–86_-specific lymphocytes following co-culture with β-endorphin and/or naloxone. Lymphocytes co-cultured with β-endorphin and/or naloxone for 72 h cells were collected and CD4, IFN-γ, IL-4, IL-17, and FoxP3 expression levels analyzed by flow cytometry. Representative results from 3 separate experiments are shown.

To examine the CD4^+^ T cell profile in rats treated with β-endorphin, lymphocytes from rats in the respective treatment groups were harvested 14 days post immunization and cultured for 6–8 h in the presence of β-endorphin. The percentage of Th1 and Th-17 cells identified in β-endorphin-treated cells was significantly lower than the levels observed from cells harvested from the EAE group. In contrast, a significant increase in Th2 cells was observed following β-endorphin treatment compared to the number observed in untreated cells (Fig. 9).

## Discussion

This report examined mechanisms associated with EA-mediated reductions in the severity of EAE in rats, in addition to examining the effects of β-endorphin (an endogenous opioid) on disease presentation. Our group demonstrated that stimulation through the Zusanli acupoint attenuated EAE severity, nevertheless rats receiving non-Zusanli acupoint therapy were still suffering serious disease. We also proved that successive eletroacupuncture treatment at the Zusanli ST36 acupoints of rats could restore the balance to the Th1/Th2/Th17/Treg T helper cell responses by stimulating the hypothalamus to increase ACTH production [20]. This is of importance since the hypothalamus is considered to be a key regulator of various physiological and pathophysiological processes including emotion, autonomic activity, and pain.

β-endorphin is an important opioid present in brain, and electroacupuncture stimulation could serve as an analgesic function by activating ACTH and/or beta-endorphin release by the brain resulting in increased hormone release [8], [23]. Gironi *et al*. demonstrated that in MS patients, β-endorphin concentrations were significantly lower than in healthy controls [1], [24]. Our research has demonstrated that MBP immunized animals developed neuropathological signs of EAE. However, EA-treated rats had markedly reduced signs of disease and demyelination, potentially due to the inhibitory effects of naloxone (Fig. 1). Furthermore, electroacupuncture stimulation promoted β-endorphin production (Fig. 6, 7, 8). These results suggested that opioids released following treatment with EA reduced the severity of EAE. In addition, Panerai *et al*. demonstrated that administration of the opiate receptor antagonist naltrexone potentiated the development of EAE in an animal model suggesting that an increase in the opioid beta-endorphin levels might represent a mechanism resulting in the down-regulation of the immune response [25]. Many studies have demonstrated that electroacupuncture possessed various therapeutic effects, including alleviation of pain, reduction of inflammation, and improvement of sleep disturbance by increasing beta-endorphin production [26]–[29].

It has been suggested that MS (defined as an autoimmune disease) is affected by imbalances between Th1 and Th2 cells. Furthermore, β-endorphin may play a role in the pathogenesis of autoimmune diseases by increasing cytokine production from the pituitary gland and lymphocytes [30]. A possible mechanism for the protective role of β-endorphin in the context of EAE may be due to changes in the cytokine expression profile. For example, β-endorphin could activate IL-4 production *in vitro* via the activation of delta-opioid receptors [31]. β-endorphin has been known to shift the Th1/Th2 balance towards a Th2 response and naloxone-mediated interference induced an increase in the Th1 cytokines IL-2 and interferon gamma and a decrease in IL-4 levels [7]. Data presented in this report also suggested that EA-mediated reduction in EAE severity was due to increased β-endorphin production that has the potential of reversing the Th1:Th2 ratio. The Th17 CD4^+^ helper T cell subset (defined by the secretion of IL-17) are considered to play an important role in promoting inflammation and autoimmunity [32], [33]. To date, this is the first report describing a role for β-endorphin on Th17 or Treg cells, and our *in vitro* results demonstrated that the percentage of Th17 cells in β-endorphin-treated cells was lower than in untreated EAE cells. Although the percentage of Tregs was not significantly different between EAE cells and β-endorphin-treated cells, we considered the possibility that in addition to β-endorphin, CRH, ACTH, and/or other substances secreted in response to EA stimulation could also have played an important role in the therapeutic effects of EA on EAE. The CD4^+^ T cell-mediated attenuation of EAE in rats was blocked in the presence of naloxone and accompanied by an increase in β-endorphin release.

The endogenous opioid peptide β-endorphin was reported to affect T lymphocyte function by either increasing proliferation or altering cytokine responses [34]–[37], inhibiting these responses [5], [38], [39], or eliciting opposing effects depending on the culture conditions [40]. For example, Garcia *et al*. found that β-endorphin inhibited in a dose-dependent manner the release of IL-2 in concanavalin A-stimulated splenic lymphocytes measured 24 h after stimulation [38] and the intracerebroventricular administration of β-endorphin induced a significant inhibition in splenocyte proliferation [39]. Recently, β-endorphin was shown to inhibit IL-2 transcription in a human T cell line [41]. In this study, proliferation of T cells harvested from EAE rats induced by the MBP_68–86_ peptide stimulation was decreased in the presence of different concentrations of BE stimulation *in vitro*; that is, BE down-regulated T cell responses. Singhal *et al*. considered that opiate-induced T cell apoptosis may be mediated through the JNK cascade and activation of caspases 8 and 3 [42].

Numerous studies have shown that EA pretreatment inhibited neuronal apoptosis in animals with cerebral diseases [43]–[45]. However, Wu *et al*. suggested that EA therapy improved ulcerative colitis in rats, likely due to the promotion of neutrophil apoptosis and the down-regulation of monocyte-derived cytokines [46]. Flow cytometric data presented in this report demonstrated that apoptosis was significantly increased in the EA group 14 and 21 days post immunization. Glucocorticoids and opioid peptides may have triggered apoptosis after binding to specific cytoplasmic membrane receptors resulting in Fas activation (resulting in apoptosis) [47].

Taken together, our recent and previous studies demonstrated that electroacupunctue treatment of rats presenting with EAE promoted the expression of β-endorphin and activated HPA to release ACTH resulting in a re-establishment of the Th1/Th2 and Th17/Treg balance and a decrease the proliferation of T-cells associated with the pathology of EAE.
